# Mechanism of Intraocular Lens Calcification After Pseudophakic Endothelial Keratoplasty

**DOI:** 10.7759/cureus.98041

**Published:** 2025-11-28

**Authors:** Panos Gartaganis, Panagiota Natsi, Sotirios Gartaganis, Petros Koutsoukos

**Affiliations:** 1 Medical Retina Service, Western Eye Hospital, Imperial College Healthcare NHS Trust, London, GBR; 2 Chemical Engineering, Laboratory of Inorganic and Analytical Chemistry, University of Patras and Foundation for Research and Technology Hellas / Institute of Chemical Engineering Sciences (FORTH/ICE-HT), Patras, GRC; 3 Ophthalmology, School of Medicine, University of Patras, Patras, GRC

**Keywords:** endothelial keratoplasty, hydrophilic intraocular lens, intraocular lens material, mechanism, opacification, pseudophakia

## Abstract

The purpose of this study is to present an experimental model that explains the calcification pattern of hydrophilic acrylic intraocular lenses (IOLs) following endothelial keratoplasty (EK) procedures using the intracameral injection of air or gas.

Pseudophakic eyes with hydrophilic acrylic IOLs undergoing EK procedures with intraocular air or gas injection are at risk of IOL calcification. Using air/aqueous humor (AH) dynamics, we attempted to explain the physicochemical mechanisms operating upon filling the anterior chamber with air (or gas) at pressures of at least 30-40 mmHg and for a period of 10-60 minutes in the presence of a hydrophilic acrylic IOL. After a short period of time, usually 10-15 minutes, the air bubble was reduced to 60-90% of the anterior chamber volume, completely covering the pupil and hydrophilic acrylic IOL surface. We have constructed a constant temperature and pressure artificial eye anterior chamber reactor (ACEACR) to simulate the anterior chamber air/gas pressure dynamics involved in applying EK surgery procedures in the presence of a hydrophilic acrylic IOL.

The analysis of the opacified IOLs in the test model showed deposits of calcium phosphate crystallites on the surface of calcified IOLs similar to clinical findings. Calcific deposits appeared as a white circular area outlining the mineralization front of the interface between the air/gas bubble and the IOL exposed in synthetic aqueous humor (SAH).

The calcification pattern of hydrophilic acrylic IOLs following EK procedures is caused by the development of locally higher calcium and phosphate concentration in comparison with the corresponding bulk AH, inside the AH meniscus formed at the air/bubble/IOL interface.

## Introduction

A rare but serious side effect of cataract surgery is intraocular lens (IOL) opacification, documented in a variety of hydrophilic acrylic IOLs as a result of calcification [1-4]. In a recent retrospective study, the rate of IOL opacification following uncomplicated phacoemulsification with various characteristics in IOLs made of different materials and designs was 0.03% [5]. Recently, minimally invasive endothelial keratoplasty (EK) procedures and their subtypes are gaining popularity as the preferred treatment for corneal endothelial dysfunction [6]. However, with the development of EK procedures, the number of IOL opacifications and ensuing explantations has increased. In the literature, rates of hydrophilic acrylic IOLs opacification following EK procedures range from 2.5% to 12.9% [7]. In all cases, the opacification exhibited a specific pattern of calcification, consisting of a more or less circular ring area on the anterior optical surface, corresponding to the zone of contact between the aqueous humor (AH) wet IOL and the injected air or gas. The calcification on the surface of hydrophilic acrylic IOLs is enhanced by locally higher concentrations of calcium phosphate species, in comparison with the surrounding AH. This locally higher supersaturation has already been shown to be the driving force for the calcification and subsequent opacification of hydrophilic acrylic IOLs [1-3]. So far, available data on the mechanism of IOL calcification development past the implementation of EK procedures are still scarce or missing, to our knowledge, in the medical literature. The purpose of this study is to contribute to the understanding of the calcification mechanism of hydrophilic acrylic IOLs following EK procedures, in which intracameral air or gas is involved. In this case, the introduction of air or gas in the anterior chamber results in confinement of the AH, resulting in the formation of an interface between the injected air or gas, the surface of the IOL, and the AH, a biological fluid supersaturated with respect to calcium phosphates [8].

## Technical report

Existing theories and concepts

Our recent laboratory investigations were concerned with the mechanisms underlying crystal formation in hydrophilic acrylic IOLs. Two main explanations for IOL calcification were suggested. First, the formation of calcium deposits was attributed to the supersaturation of AH with respect to a number of crystalline calcium phosphate phases. These phases include, besides hydroxyapatite (Ca_5_ (PO_4_)3OH, or HAP), dicalcium phosphate dihydrate (CaHPO_4_·2H_2_O, DCPD), and octacalcium phosphate (Ca_8_H_2_(PO_4_)_6_·5H_2_O, OCP), which have been identified in normal and pathological clinical findings [1-3,9]. This condition is favourable for the precipitation of the respective salts.

Second, we need to consider an additional important factor involved in the pathophysiology of this problem, which is related to the presence of hydroxyl groups on the surface of the polyacrylic materials used in the fabrication of hydrophilic acrylic IOLs. The hydroxyl groups and the polar carbonyl groups of the poly 2-hydroxyethyl methacrylate (PHEMA) matrix favor the formation of surface complexes with the bivalent calcium ions. The respective surface sites facilitate nucleation and further growth of calcium phosphates [1-3].

The water content of the IOLs is an important factor, which explains why hydrophobic IOLs do not calcify. The latter IOLs, made as a rule of poly-methyl methacrylate (PMMA), have low water content, between 0.5% and 4% [10]. Hydrophilic acrylic IOLs, often fabricated of PHEMA polymer, or a blend of 2-hydroxyethyl methacrylate and methyl methacrylate, contain water in the range of 18%-38% [10]. Hydroxyl and esterified carboxyl functional groups of the IOL polymeric matrix determine the surface charge of the material through protonation/deprotonation reactions. Higher water content enhances the ionization of the functional groups on the polymer surface, forming surface complexes with AH calcium ions [1,2]. When air or gas is pressurized against hydrophilic acrylic IOLs (as occurs during endothelial keratoplasty), it creates forces at the lens surface that push water molecules away from the IOL material. This process, called disjoining pressure, disrupts the normal water layer on the lens.

As a result, the AH in that local area becomes supersaturated with calcium and phosphate, meaning it contains more of these minerals than it can normally hold in solution (this supersaturation is like adding too much sugar to water until it can no longer dissolve).

The combination of the disrupted water layer and the mineral-rich environment creates ideal conditions for calcium phosphate crystals to form and grow on the IOL surface, leading to lens opacification.

Methodology

Recently, an increasingly higher number of reports regarding the postoperative opacification of hydrophilic acrylic IOLs after EK procedures have been published [11-15]. In procedures like Descemet membrane endothelial keratoplasty (DMEK), where a corneal graft including Descemet’s membrane and endothelium is transplanted, injecting air or gas, forming a bubble into the anterior chamber, helps ensure graft adhesion to the posterior corneal surface. The air or gas bubble provides a tamponade, keeping the graft in place and facilitating its attachment to the corneal tissue. Ideally, an air/gas bubble should form inside the eye, filling about 90% to 100% of the anterior chamber. The air or gas bubble in the anterior chamber at the end of the procedure must move freely. Thereafter, the bubble encompasses the entire pupil and the hydrophilic acrylic IOL surface. In the present work, only air was tested.

**Figure 1:**
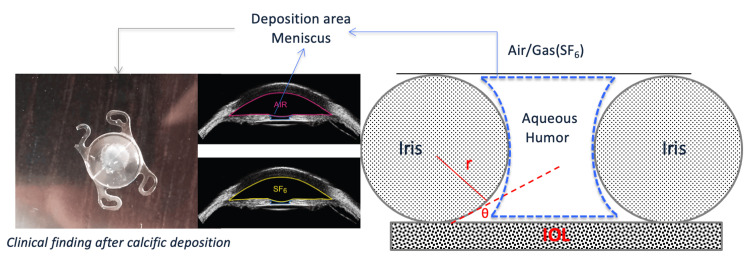
Schematic illustration of aqueous humor meniscus between the air/gas bubble and the anterior surface of the IOL Graphical outline of the concept of IOL calcification associated with DMEK described in the paper. The AH meniscus formed by applying the air or gas on the IOL is a domain of higher supersaturation with respect to HAP in comparison with that of the bulk solution, leading to the formation of calcific deposits along the circumference of the bubble in contact with the IOL. IOL: intraocular lens; DMEK: Descemet membrane endothelial keratoplasty; AH: aqueous humor; HAP: hydroxyapatite Original figure created by the authors

It is suggested that the formation of an AH meniscus between the bubble and the anterior surface of the hydrophilic acrylic IOL is a possible causative mechanism of the calcification pattern found following EK procedures. Considering the topology of AH meniscus formation, the fluid element characteristics depend on the meniscus curvature radius (r) and the contact angle (θ) between the posterior surface of the iris and the anterior surface of the hydrophilic acrylic IOL. To simplify the calculations, it was assumed that the meniscus is spherical (Figure 1). Local supersaturation of the AH within the meniscus is the driving force for the formation of hydroxyapatite (Ca_5_(PO_4_)3OH, HAP), which may take place either directly or through the transformation of less stable transient calcium phosphate phases. The formation of a stable thin AH film (meniscus) on the hydrophilic acrylic IOL surface is critical. Due to the confinement of the AH element by curved surfaces, the concomitant supersaturation with respect to HAP is higher in comparison to the corresponding bulk AH, considering the disjoining pressure at the gas/polymer/AH interface [16,17]. This effect is the thermodynamic driving force needed for the formation of HAP crystallites found in clinical cases of IOL opacification due to calcification [18,19].

Model experiments

**Figure 2:**
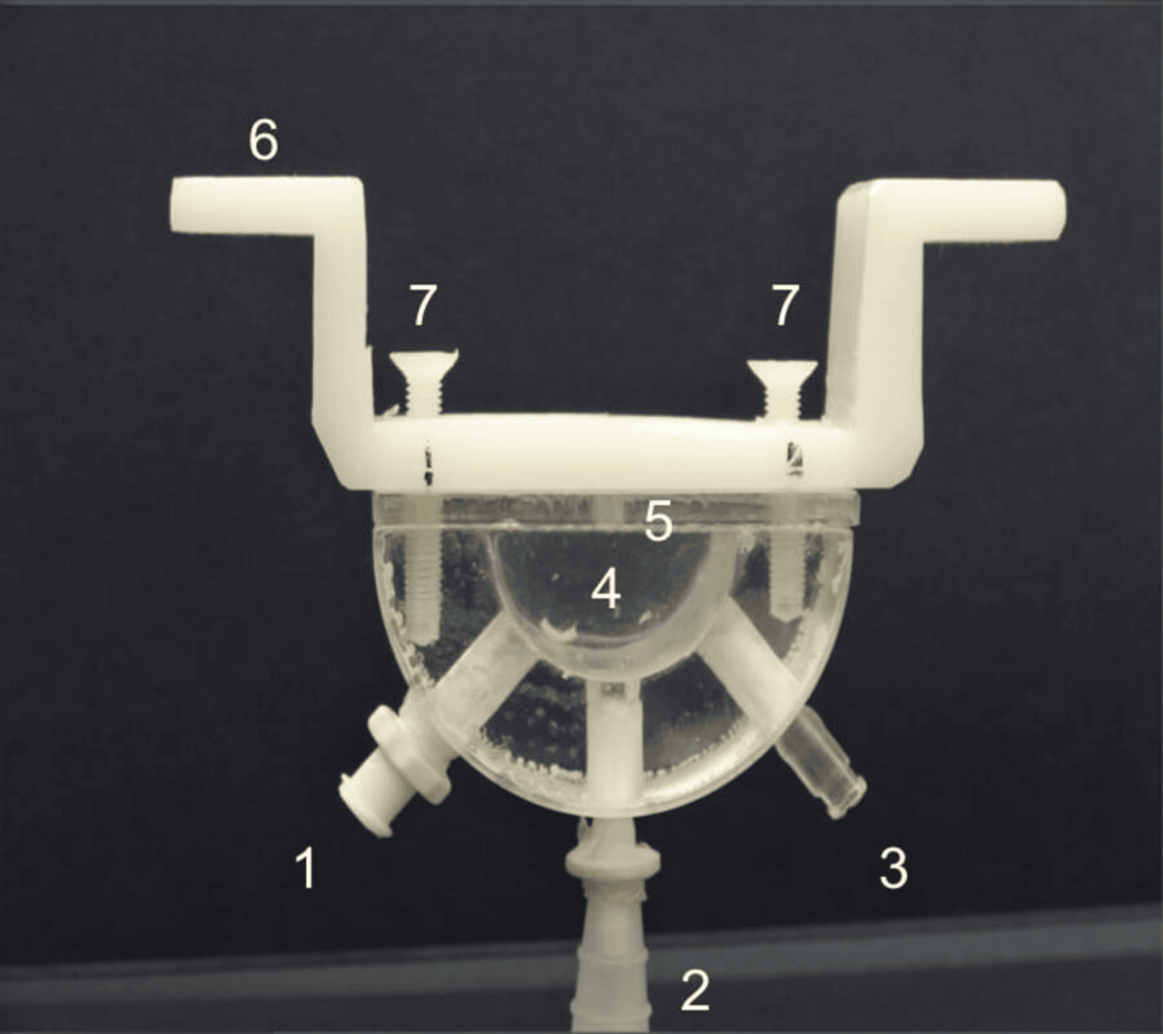
Photograph of artificial eye anterior chamber reactor (ACEACR) Original image of the experimental model, constructed in our laboratory, to simulate and validate the concept described in Figure 1. (1) Port for air/gas injection, (2) Port for filling vessel 4 with SAH, (3) Fluid outlet for the regulation of air/gas pressure and connection port for manometer to measure pressure, (4) Vessel containing SAH, (5) IOL position between cell and holder, (6) Holder for immersion of the cell in a water bath thermostated at 37 °C, (7) Screws to tighten IOL for contact with SAH contained in vessel 4. The distance between the screws at positions 7 and 7 is 4.5 cm. SAH: synthetic aqueous humor; IOL: intraocular lens

A cell was designed and constructed to simulate the process of AH meniscus formation, allowing the formation of calcific deposits at the air/simulated aqueous humor (SAH)/hydrophilic acrylic IOL interface. The stability of SAH at the interface with air/gas on hydrophilic acrylic IOLs was studied. The construction of a constant temperature and pressure artificial eye anterior chamber reactor (ACEACR) for simulating air/gas pressure was used to simulate the DMEK surgery, in the presence of a hydrophilic acrylic IOL, as indicated by the literature (Figure 2).

The cell was thermostated at 37 °C (Haake Circulating Thermostat, Thermo Fisher Scientific, Waltham, MA, US), and the hydrophilic acrylic IOLs were immersed in SAH, which was supersaturated with respect to calcium phosphate. The SAH was pressed out of the chamber, leaving a film of 5.0-5.5 mm diameter and an estimated 1 mm thickness on the exposed part of the hydrophilic acrylic IOL. A constant pressure of around 30 mmHg was maintained throughout and monitored through the appropriate manometric sensors (physiological pressure transducer (ADInstruments, Dunedin, New Zealand)). The duration of the experiments ranged from 5 to 60 minutes. Four hydrophilic IOLs were used in respective experimental runs. Past residence of the air bubble on the test IOL was from 10 to 60 minutes (10, 20, 40, and 60 minutes); the same calcification pattern was observed, ensuring high reproducibility of the obtained results both in terms of the nature of the calcific deposits and the pattern of the crystalline deposits. Calcified deposits were detected by optical microscopy (Olympus Corporation, Hachioji, Tokyo, Japan) and scanning electron microscopy (SEM) investigation (Zeiss, LEO VP-35 FEM (Zeiss Group, Oberkochen, Germany), equipped with a Bruker AXS microanalysis unit; Bruker, Billerica, MA, US).

Experimental results

Calcification at the Air-SAH-IOL Interface

**Figure 3:**
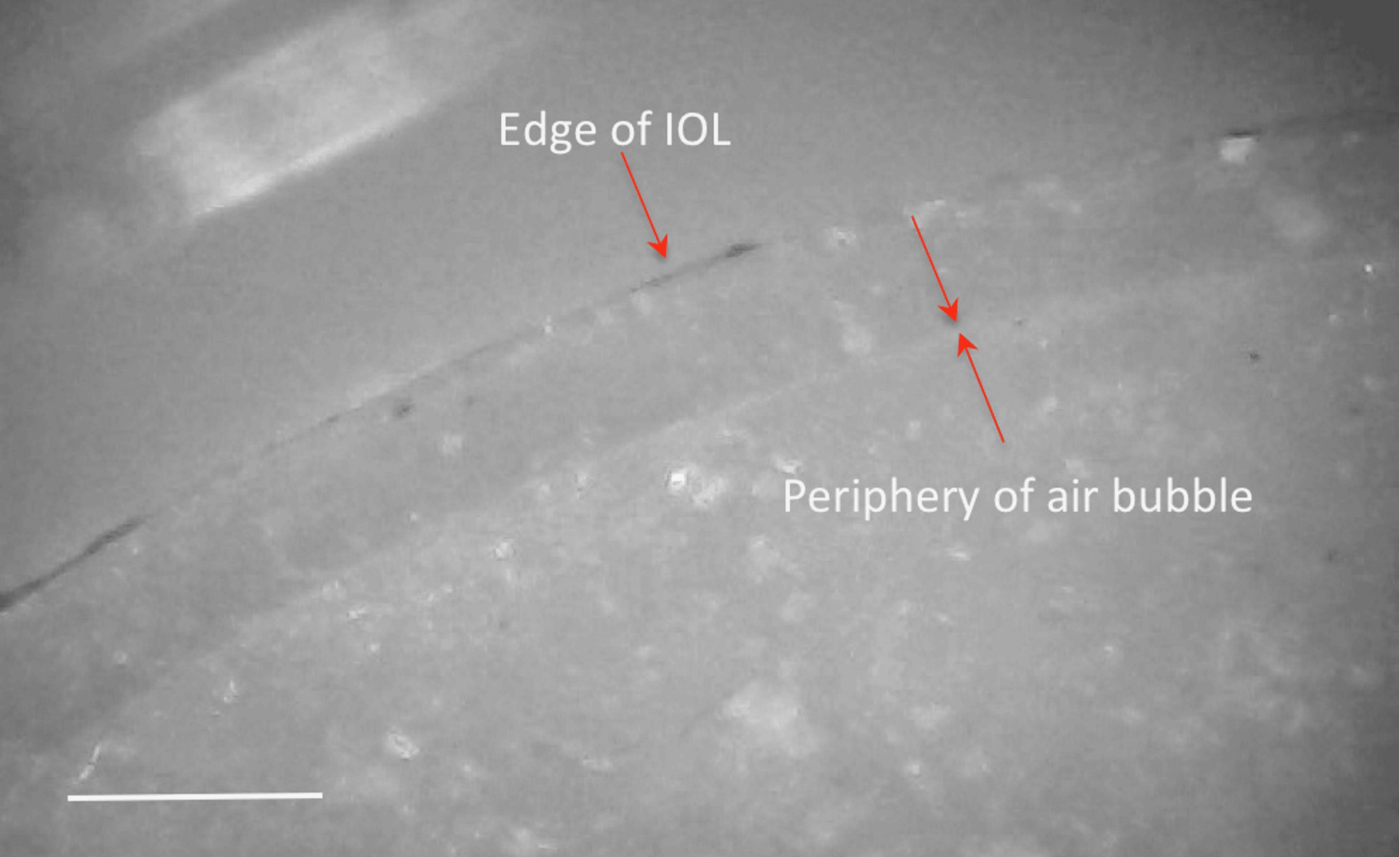
Light microscopy image showing the calcification pattern of the IOL after 24 hours of air-bubble exposure Optical microscopy image acquired using an Olympus microscope owned and operated by the laboratory(ies) in which the experiments were conducted. Areas of the anterior surface of the exposed IOL in which numerous and confluent rectangular and spherical calcium deposits are present demonstrate the spread of calcific deposits initiated at the gas/SAH/IOL interface. Scale bar size=100 μm SAH: synthetic aqueous humor; IOL: intraocular lens

**Figure 4:**
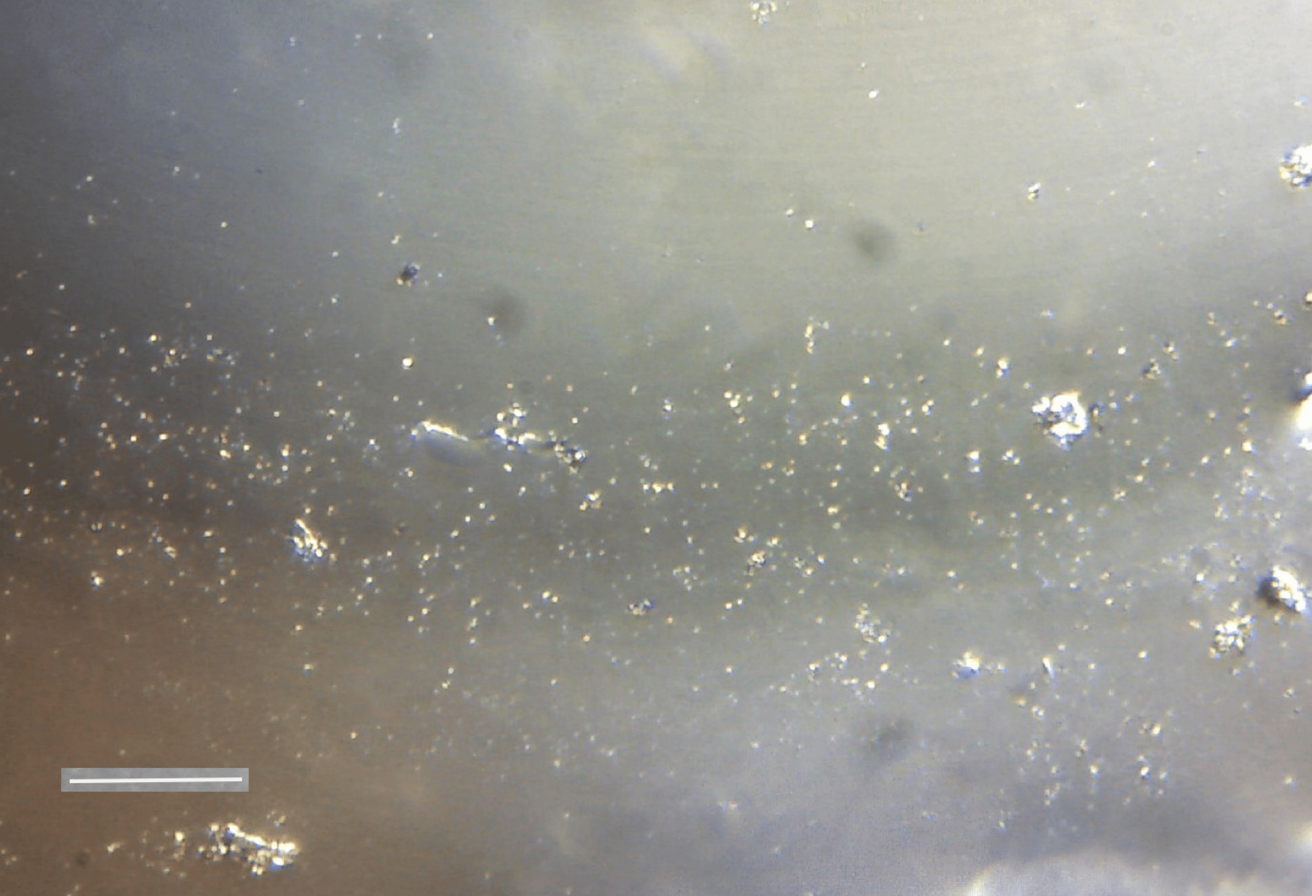
Light microscopy image showing pronounced peripheral calcification at the gas/SAH/IOL interface Optical microscopy image acquired using an Olympus microscope owned and operated by the laboratory(ies) in which the experiments were conducted. The image shows part of the calcific deposits formed along the periphery of the air bubble in contact with the test hydrophilic IOL. Scale bar size=100 μm SAH: synthetic aqueous humor; IOL: intraocular lens

Details of the calcified deposits were examined by light microscopy examination on the exposed hydrophilic acrylic IOLs on which an air bubble of diameter 5.5 mm was formed and remained in contact with the hydrophilic acrylic IOL for 24 hours. The formation of the calcific deposits was monitored for the first 60 minutes. A whitish circular ring indicates the front of mineralization at the interface between the air bubble, the IOL exposed, and the SAH at 37 °C (Figures 3, 4). The composition of SAH, which contained only inorganic components, is identical to that of electrolyte concentrations in normal AH, where the precise concentrations are as follows: sodium (Na^+^) = 146 μmol/mL, potassium (K^+^)= 5.25 μmol/mL, calcium (Ca^++^) = 1.70 μmol/mL, magnesium (Mg^++^) = 0.8 μmol/mL, chloride (Cl^-^) = 109.5 μmol/mL, bicarbonate (HCO^3-^) = 33.6 μmol/mL, and phosphate (H_2_PO_4_) = 0.62 μmol/mL as described by Drimtzias et al. [9].

**Figure 5:**
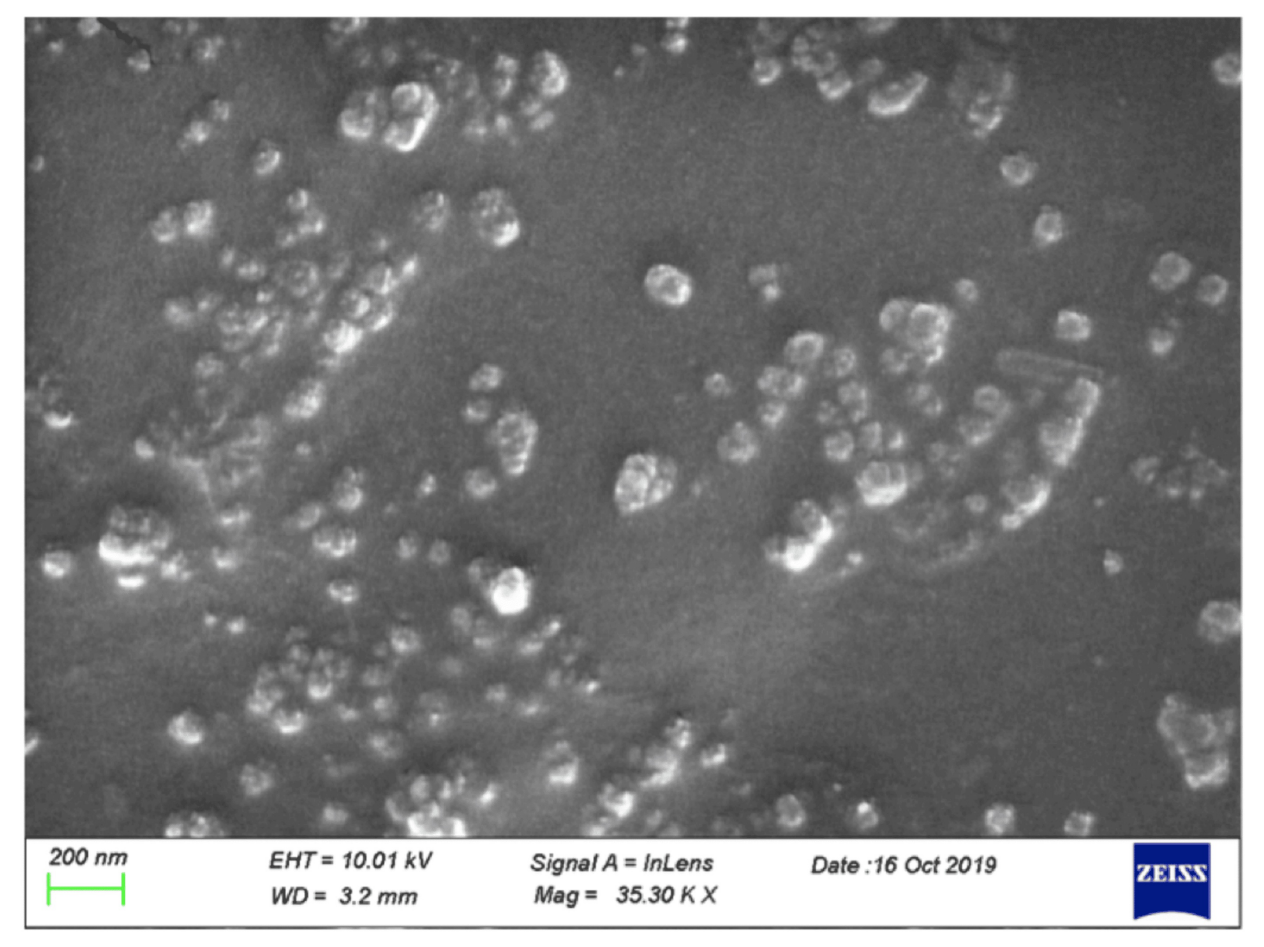
SEM image of the anterior surface of the opacified hydrophilic acrylic IOL SEM image acquired using a Zeiss LEO VP-35 FEM platform equipped with a Bruker AXS microanalysis unit owned and operated by the laboratory(ies) in which the experiments were conducted. The surface texture of the deposits on the IOL shows lumps containing salts outgrowing from the interior of the polymeric IOL material, scale bar size=200nm. The breadth of the periphery of the calcific deposits is ca. 10 microns. SEM: scanning electron microscopy; IOL: intraocular lens

**Figure 6:**
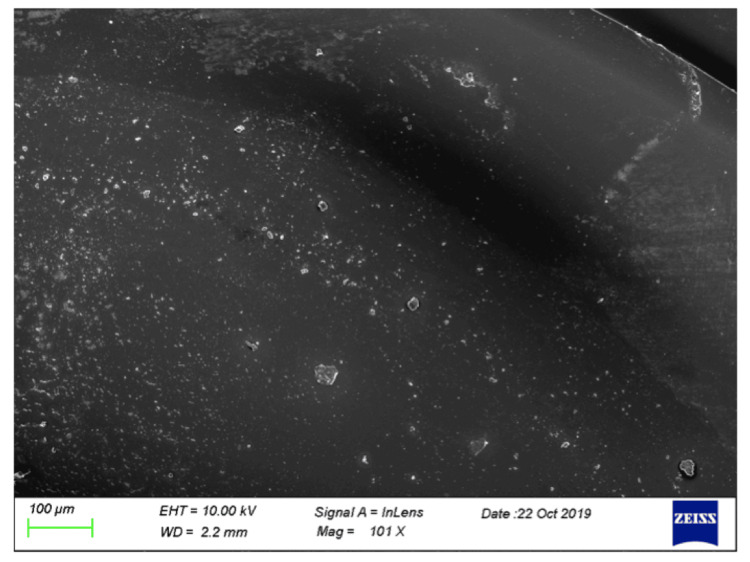
Calcific deposits formed upon exposure of hydrophilic acrylic IOL in SAH with an air bubble on its surface for 24 hrs. SEM image acquired using a Zeiss LEO VP-35 FEM platform equipped with a Bruker AXS microanalysis unit owned and operated by the laboratory(ies) in which the experiments were conducted. The calcific deposits spread from the periphery of the bubble-air interface toward the central part of the lens, suggesting that the starting point Is the gas/IOL interface and that the formation of calcific deposits continues from this point on. Scale bar size=100 μm SAH: synthetic aqueous humor; IOL: intraocular lens; SEM: scanning electron microscopy

Examination of the deposits by SEM revealed that the deposits appeared as lumps of solid calcium phosphate outgrowing from the interior of the hydrophilic acrylic IOL material (Figures 5, 6).

## Discussion

Since the development of EK procedures in 1998 [6], significant advances have been made in improving visual acuity and decreasing the rate of complications. However, a relatively new complication after EK procedures has emerged, which is IOL opacification. Based on literature reports, IOL opacification past EK procedures occurs only with hydrophilic acrylic IOLs and is attributed to calcium salt deposition [11-15].

Physicochemical changes at the air/gas and hydrophilic acrylic IOL interface due to the presence of the AH meniscus may lead to changes in the supersaturation of the AH with respect to calcium phosphates and cause the formation of calcific deposits on the surface and/or in the interior of IOLs. Local supersaturation is the driving force for HAP formation, which can take place either directly from the AH solution or through the transformation of less stable transient calcium phosphate phases (e.g., OCP). 

Local supersaturation depends on the curvature of the meniscus developed at the air/IOL interface. In this case, the main limitation of the methodology proposed for the investigation of calcification in EK processes is the size of the air bubble attached to the IOL and the persistence of the stability of the bubble for sufficiently long times to study the evolution of the calcification process. It is desirable to extend the residence time of the air bubble for long time periods comparable to those encountered in clinical practice.

The locally developed supersaturation on the IOL surface due to contact with the emerging AH meniscus is inversely proportional to the curvature of the AH meniscus. A small circle radius (Figure 1) corresponds to high curvature, resulting in a small meniscus. The supersaturation with respect to calcium phosphates in the AH confined in this meniscus is locally higher in comparison to that of the bulk AH, resulting in a higher probability of nucleation and subsequent crystal growth of the calcium phosphate forming (presumably HAP). On the other hand, a larger circle radius (Figure 1) corresponds to low curvature, where the local supersaturation is closer to that in the bulk AH. A change of the radius of curvature by 10% changes the solution supersaturation by more than 12% as may be calculated from the Young-Laplace equation [17,18]. The local supersaturation due to the formation of the air or gas/ΑΗ/IOL interface depends on the type of gas used in EK procedures. Air contains O_2_, N_2_, and relatively significant concentrations of CO_2_. In the case of air, the meniscus formed a larger radius bubble corresponding to lower curvature in comparison with the meniscus predicted for gas bubbles of SF6. SF6 gas is hydrophobic, and this results in the reduction of the meniscus radius corresponding to an overall higher curvature. It is anticipated that local supersaturation of the AH with respect to HAP in the case of air is lower in comparison to that formed by SF6. Air composition (O_2_, N_2_, and CO_2_) implies strong interaction with aqueous electrolyte solutions because of the solubility of CO_2_ and O_2_ in water. On the contrary, SF6, a completely non-polar, water-insoluble gas, is expected to have practically no chemical interaction with water, resulting in a higher radius of the curvature of the meniscus formed upon contact of the gas with the IOL. Current work on the type of gas used is ongoing.

The calcific formations developed on hydrophilic acrylic IOLs following EK procedures have mostly been described as a more or less circular area of the anterior optical surface, corresponding to the zone of contact with the injected air or gas [11-13]. Particles focused primarily at the circumference tend to spread toward the central part of the IOL.

The IOL mineralization experiments, performed in our in vitro model, confirmed that the deposits' pattern and size mimic the shape of the AH meniscus we described, which corresponds to the optical zone of the intraocular lens and is surrounded by the capsulorhexis and wetted by the AH. The pathogenetic mechanism leading to hydrophilic acrylic IOL calcification consists of the fact that the ionized surface groups (O^-^) of the polyacrylic materials are key factors catalyzing surface nucleation and growth of calcium phosphate from supersaturated solutions. The calcium and phosphate species diffuse into the hydrophilic acrylic IOL polymer, eventually promoting nucleation and crystal growth of HAP in the interior of the IOL. Furthermore, in our opinion, surface calcification of hydrophilic acrylic IOLs during EK procedures, as the anterior chamber fills with air or gas, is enhanced through the development of a locally higher supersaturation in comparison with the conditions reported earlier, which were, however, sufficient to cause calcification of the hydrophilic acrylic IOLs from the bulk aqueous fluid [1-3,9].

The thermodynamic driving force of the AH meniscus at the air/gas interface of the IOL's anterior surface also depends on the temporal difference between the meniscus's presence in the anterior chamber, which varies between air and gas. The air bubble will be dissipated in 3 to 4 days, and the 20% SF6 gas can last from 6 to 8 days, allowing for the presence of the gas in the anterior chamber for longer time periods [20]. Furthermore, an additional variable affecting the thermodynamic driving force of the AH meniscus is the variable time period for postoperative maintenance of supine position, ranging from 2 to 4 hours, and up to 12 hours daily.

Hydrophilic IOLs are more susceptible to calcification, which can have a significant impact on vision. Consequently, they are not the optimal choice for patients with corneal or posterior segment disorders who may require future procedures involving intraocular air or gas. Avoid using hydrophilic acrylic IOLs in patients with endothelial diseases who are likely to require future procedures that involve intraocular air or gas such as EK.

This experimental model has the potential to study how different risk factors affect this condition and to identify which hydrophilic IOLs have the greatest propensity to calcify. The model can also serve as an educational tool to help us better understand this pathology.

Finally, hydrophilic acrylic IOL calcification is a rare postoperative complication and, based on evidence, will guide future research on IOL calcification following EK procedures, the development of calcium deposit removal treatments, and the investigation of calcification protection for hydrophilic acrylic IOLs.

## Conclusions

This report proposes a mechanistic explanation for calcification patterns observed in hydrophilic acrylic IOLs following EK procedures. When gas bubbles contact the IOL surface, the curved AH meniscus creates a confined space where disjoining pressure may locally increase calcium phosphate concentrations beyond normal levels.

This localized supersaturation can promote the nucleation of HAP crystallites, which appear as nanoparticles that tend to aggregate. The calcific deposits characteristically develop at the circumference where the gas bubble contacts both the IOL and AH, consistent with this proposed mechanism.
